# Magnetic Modulation of Biochemical Synthesis in Synthetic Cells

**DOI:** 10.1021/jacs.4c00845

**Published:** 2024-05-01

**Authors:** Karen K. Zhu, Ignacio Gispert Contamina, Oscar Ces, Laura M. C. Barter, James W. Hindley, Yuval Elani

**Affiliations:** † Department of Chemistry, Imperial College London, Molecular Sciences Research Hub, White City, London W12 0BZ, U.K.; ‡ Department of Chemical Engineering, Imperial College London, South Kensington, London SW7 2AZ, U.K.; § fabriCELL, Imperial College London, Molecular Sciences Research Hub, White City, London W12 0BZ, U.K.; ∥ Institute of Chemical Biology, Imperial College London, Molecular Sciences Research Hub, White City, London W12 0BZ, U.K.

## Abstract

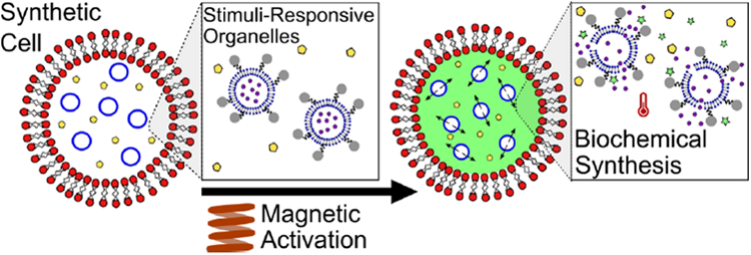

Synthetic cells can be constructed from diverse molecular components, without the design constraints associated with modifying 'living' biological systems. This can be exploited to generate cells with abiotic components, creating functionalities absent in biology. One example is magnetic responsiveness, the activation and modulation of encapsulated biochemical processes using a magnetic field, which is absent from existing synthetic cell designs. This is a critical oversight, as magnetic fields are uniquely bio-orthogonal, noninvasive, and highly penetrative. Here, we address this by producing artificial magneto-responsive organelles by coupling thermoresponsive membranes with hyperthermic Fe_3_O_4_ nanoparticles and embedding them in synthetic cells. Combining these systems enables synthetic cell microreactors to be built using a nested vesicle architecture, which can respond to alternating magnetic fields through in situ enzymatic catalysis. We also demonstrate the modulation of biochemical reactions by using different magnetic field strengths and the potential to tune the system using different lipid compositions. This platform could unlock a wide range of applications for synthetic cells as programmable micromachines in biomedicine and biotechnology.

## Introduction

Biological cells can be viewed as complex microreactors that enable a multitude of reactions necessary for life to occur in parallel. These reactions include those required for metabolism, gene expression, and protein production. Inspired by this, in the field of synthetic biology, researchers seek to build synthetic cells which can be used to aid our understanding of individual processes in living cells while also enabling bespoke pathways to be engineered for designated purposes and applications, including drug delivery , in vitro cell models, and microreactors for bioproduction.^1,2^ Analogously to living cells, synthetic cells are often bound by a lipid membrane and use liposomes as an architectural motif. Much of the recent focus within the field has been on incorporating increasing degrees of compartmentalization into these liposomal structures.^3^ This compartmentalization can be used to create spatial separation, which, when applied in conjunction with stimuli-responsive membranes, can also allow for spatiotemporal activation and the development of synthetic communication pathways.^4−8^

External control of synthetic cells via stimuli-responsive systems allows for remote activation of cellular processes. This is especially useful in biomedical settings, including in targeted therapeutics. It has also been used in engineering biology more broadly, where it allows biological cells to respond to complex physical triggers, using synthetic cells as intermediaries instead of direct genetic engineering.^8^ Two classes of stimuli can be employed: chemical (e.g., pH and chemical inducers)^9−12^ or physical (e.g., temperature and light).^7,8,13−16^

Physical stimuli offer a noninvasive and precise approach for remotely controlling engineered processes, enabling quick responses with high spatiotemporal accuracy, and reducing toxicity and biological interference. Over the past decade, several examples of both light- and temperature-responsive systems have been reported in synthetic cell microreactors.^7,8,13,17−22^ To date, however, magnetic activation of synthetic cells has not been achieved.

The use of magnetic fields as a physical trigger is attractive due to their ability to noninvasively penetrate through tissue, with low toxicity and intrinsic biorthogonality, allowing for long-range, spatially controlled activation.^23,24^ Magnetic nanoparticles which are controlled by magnetic fields currently have a wide range of biomedical and biotechnological applications, including magnetic resonance imaging (MRI),^3,25^ protein biodetection,^26−28^ antimicrobial applications,^29−31^ hyperthermia treatments,^32−36^ and nanorobotics.^37,38^ However, the dependence of magnetic systems on abiotic/inorganic building blocks, as opposed to protein and nucleic acid machinery, hinders their exploitation in top-down synthetic biology (where living cells are genetically engineered to have new capabilities), although progress is being made.^39^

**Figure 1:**
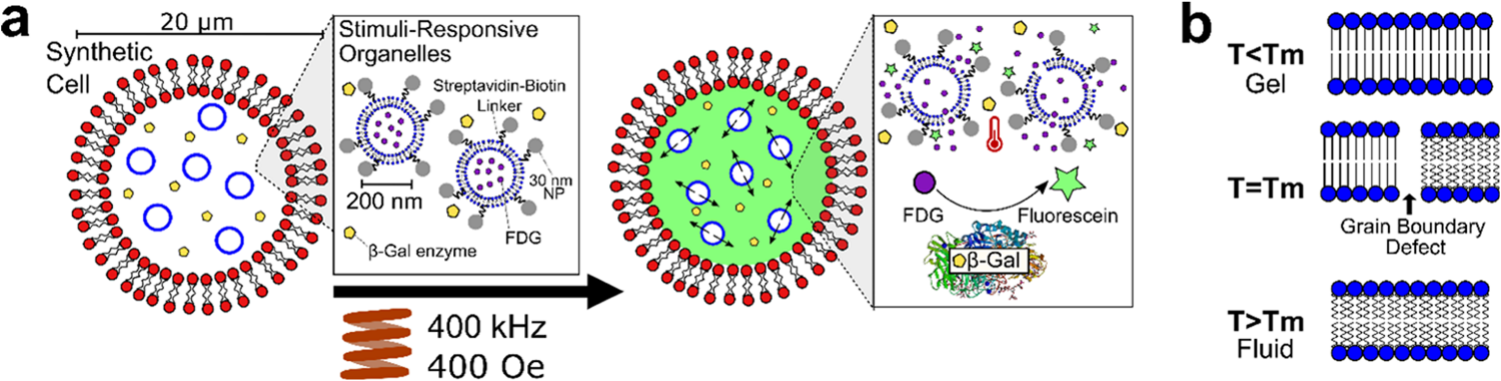
(a) Schematic showing POPC synthetic cells containing 200 nm DMPC synthetic stimuli-responsive organelles conjugated with magnetic nanoparticles, which in turn contain encapsulated fluorescein di-β-d-galactopyranoside (FDG) (nonfluorescent). FDG is released upon application of the magnetic field (400 Oe) due to local heating of the organelle membrane and subsequent phase transition. This leads to FDG hydrolysis and the production of fluorescein (fluorescent) by action of the enzyme β-Gal within the lumen of the synthetic cell. (b) Schematic showing the mechanism of content release from the organelles. Grain boundary defects form at the phase transition temperature (*T*_m_) of a lipid bilayer, as sections of the membrane melt into the fluid phase, while others remain in the gel phase, allowing for transport of the cargo across the membrane.

This can be remedied using a bottom-up synthetic approach which is not constrained by the building blocks of biology; building cells from scratch allows us to incorporate alternative molecular components with ease, including magnetic nanoparticles. Previous research used Au nanocrystals with magnetic induction heating to control DNA hybridization;^40^ however, we are aware of no applications in the context of synthetic cells. Here, we constructed multicompartment synthetic cell microreactors, which exploit synthetic organelles composed of engineered thermosensitive membranes and Fe_3_O_4_ magnetic nanoparticles for magnetic response functionality. These microreactors can produce a fluorescent product in response to an alternating magnetic field via nanoparticle-mediated induction heating of thermoresponsive organelles, which in turn causes substrate release from the organelles into the cell lumen and initiation of enzymatic biocatalysis (Figure 1a). Importantly, this system is dormant until stimulus activation, facilitating a high degree of temporal control over synthetic cell function. When combined with the noninvasive and deep tissue penetration characteristics of magnetic fields, this design approach can be adapted for multiple applications including in vesicle bioreactors, therapeutic delivery, and components of synthetic cell communication pathways, by altering the membrane compositions and the encapsulated cargo.

## Results and Discussion

Lipids undergo phase changes in response to temperature, with the gel–fluid transition being the most important for the principles of this experiment. At the transition temperature (*T*_m_), grain boundary defects form between portions of the membrane that have melted and those that remain in the gel phase, which cease to exist once the membrane fully melts and becomes homogeneous again (Figure 1b).^41,42^ To create a triggerable system, the internal 200 nm synthetic organelles must have a gel–fluid phase transition temperature above the ambient experimental temperature (∼18 °C), while being within the temperature range that the nanoparticles are able to heat. The lipid composition of the outer giant unilamellar vesicle (GUV), which makes up the membrane of the synthetic cell, on the other hand, must be stable within this whole temperature range. In the experiments reported here, it was possible to increase the bulk temperature to 37 °C by induction heating of the Fe_3_O_4_ nanoparticles in solution, when not encapsulated in GUVs or bound to any membrane (SI Figure 1). It is expected that the surface temperature of these nanoparticles is considerably higher, with the exact extent of heating dependent on many variables that are unique to each nanoparticle.^33,34,43^ Previous studies have shown that the dissipation of heat from the surface of the nanoparticle occurs over nanometer distances.^44,45^ The nanoparticles in this experiment, at 30 nm size, are superparamagnetic iron oxide nanoparticles (SPIONs) and are biocompatible.^23,46,47^ This size was chosen as larger nanoparticles are known to allow for larger degrees of induction heating but, are not limited to remain within the superparamagnetic regime. This superparamagnetic state is greatly desirable as it prevents nanoparticles from being attracted to each other and thus minimizes agglomeration.^48−50^

1,2-Dimyristoyl-*sn*-glycero-3-phosphocholine (DMPC or 14:0 PC) with a phase transition temperature of 24.1 °C^51^ was chosen to be the primary component of the 200 nm synthetic organelles shown in Figure 1a. Biotinyl-capped lipids were added to the DMPC to establish biotin–streptavidin linkers to the Fe_3_O_4_ nanoparticles, which were coated in streptavidin, thereby increasing the encapsulation efficiency of the nanoparticles in GUVs and ensuring the proximity of the nanoparticles to the thermoresponsive organelle membrane. 1-Palmitoyl-2-oleoyl-glycero-3-phosphocholine (POPC) was chosen to be the sole component of the outer GUV as it has a phase transition temperature of ∼−3 °C,^52,53^ preventing the formation of grain boundary defects within this membrane during heating/cooling. The induction heating system setup can be seen in the Supporting Information (SI Figure 2).

**Figure 2:**
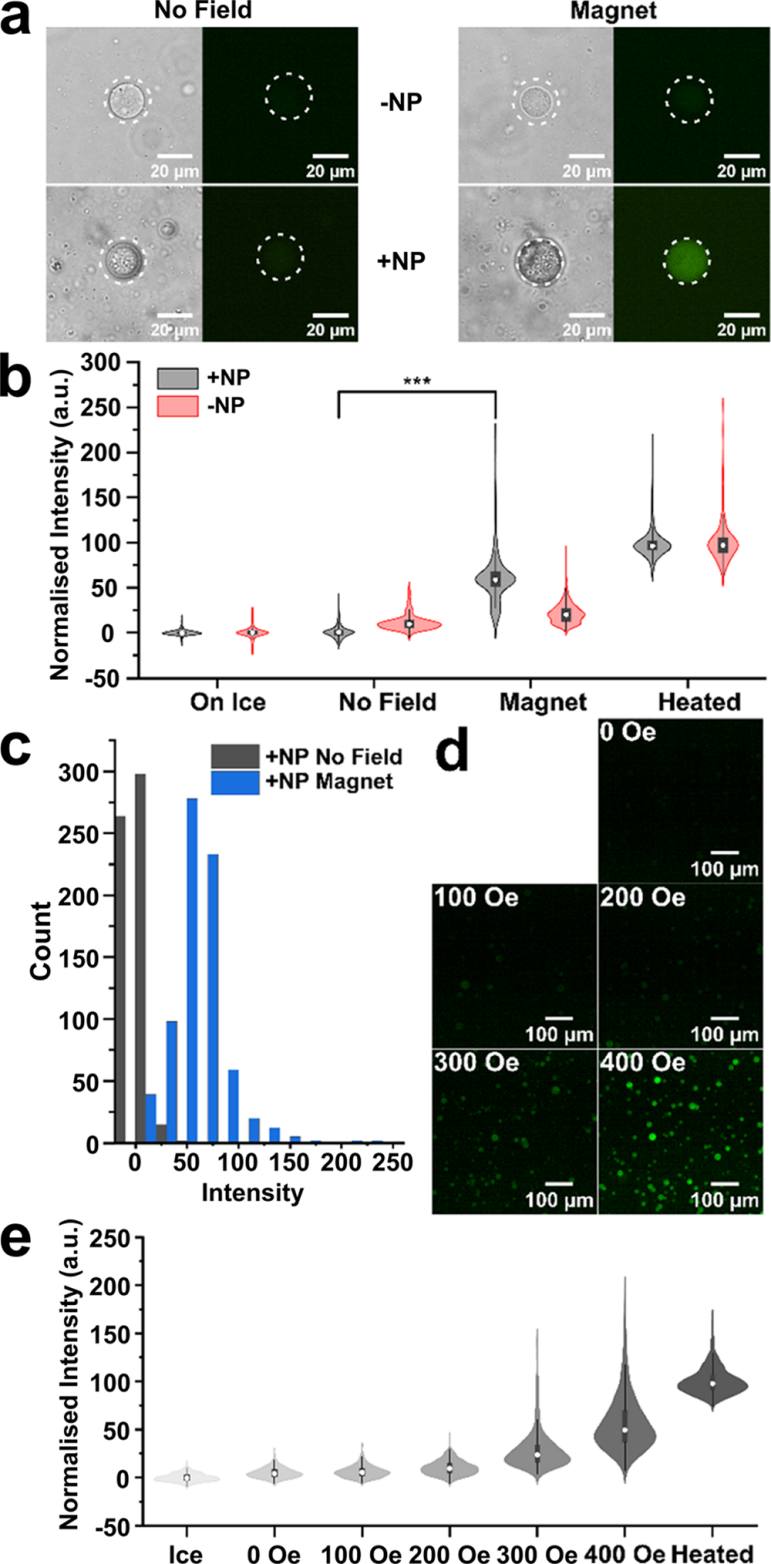
(a) Representative microscopy images showing the changes in fluorescence (produced by fluorescein from the hydrolysis of the released FDG by β-gal) upon application of the magnetic field with and without the inclusion of magnetic nanoparticles within the synthetic cell. (b) Violin plot showing the distribution in fluorescence intensities obtained from microscopy images of over 200 synthetic cells in each condition. The complete distribution of all the synthetic cells exposed to each condition is shown with the black boxes representing the interquartile range (IQR), the white dot showing the median, and the whiskers 1.5 IQR. Significance data were calculated using a two-tailed Welch’s *t* test (*** = *p* < 0.01). (c) Histogram distribution of the intensities obtained from the fluorescence microscopy images, showing the distinct difference in population fluorescence between samples exposed to the magnetic field with and without magnetic particles. (d) Representative fluorescence microscopy images of synthetic cells with different magnetic field strengths. (e) Violin plot showing the distribution in fluorescence intensities obtained from microscopy images of over 500 synthetic cells after exposure to varying magnetic field strengths.

The hydrolysis of fluorescein di-β-d-galactopyranoside (FDG) by the enzyme β-galactosidase was employed in this study as a model system for biochemical synthesis as the FDG substrate is smaller than the size of the grain boundary defects within the membrane (cutoff between 900 Da^54^ and 1674 Da^16^; ∼10 nm^41,54^). 200 nm synthetic organelles containing FDG were formed via the thin-film hydration method, followed by extrusion (SI Figure 3), and purified via a size exclusion column, before being mixed in an equal ratio with streptavidin-coated Fe_3_O_4_ nanoparticles. The nanoparticle-bound 200 nm synthetic organelles were then encapsulated with β-galactosidase within a synthetic cell via the well-established emulsion phase transfer (EPT) technique (Figure 2a).^51^ These synthetic cells form volumes with high concentrations of nanoparticles and thermoresponsive membranes, encouraging efficient induction and heat transfer. This enables localized heating to occur within the synthetic cells without noticeably increasing the bulk temperature of the sample (SI Table 1).

The complete system was exposed to an alternating magnetic field of 400 Oe strength for 15 min before being left to stand on ice for a further 30 min. Samples were then transferred to a microscope with the fluorescence intensity of the synthetic cells extracted from the microscopy images. In Figure 2b, over 200 synthetic cells were analyzed from population-representative microscopy images of each condition (SI Figures 4 and 5) to show that there were statistically significant (calculated using a two-tailed Welch’s *t* test, *p* < 0.01) increases in localized fluorescence observed between the samples with nanoparticles and exposed to the magnetic field (black) compared to those without the nanoparticles or those that were not exposed to a magnetic field (red). This can also be seen in Figure 2c when directly comparing the effect of the magnetic field with a clear shift of the peak toward higher intensity values upon application of the magnetic field (black to blue), indicating that our assembled synthetic cell could activate biosynthesis in response to an external magnetic field. Finally, heating to 40 °C also resulted in an increase in sample fluorescence, confirming that system activation occurs through temperature-induced content release. Additional experiments were performed over a range of magnetic field strengths to determine whether modulation of the release was possible. As shown in Figure 2d,e, at low magnetic field strengths of 100 and 200 Oe, a very little increase in fluorescence was obtained compared to that of 0 Oe and so it can be assumed that little to no release of FDG from the 200 nm organelles was observed. At 300 Oe, a more prominent increase in fluorescence was seen, showing that at 300 Oe, the release of FDG from some of the organelles occured. An even larger increase was observed at 400 Oe compared to 300 Oe, showing even more release of FDG. This difference in the amount of FDG release can be attributed to the proportion of organelles undergoing phase transitions, thus producing grain boundary defects, in each sample of synthetic cells. It can be assumed that at 400 Oe, a larger proportion of organelles were able to reach temperatures that would cause a phase transition compared to those at 300 Oe. At 300 Oe, the variation in release from the organelles may be due to variations in the nanoparticles themselves, as well as variations in the number of nanoparticles near each organelle, and the degree of proximity of each nanoparticle to the membrane of the organelles.

**Figure 3:**
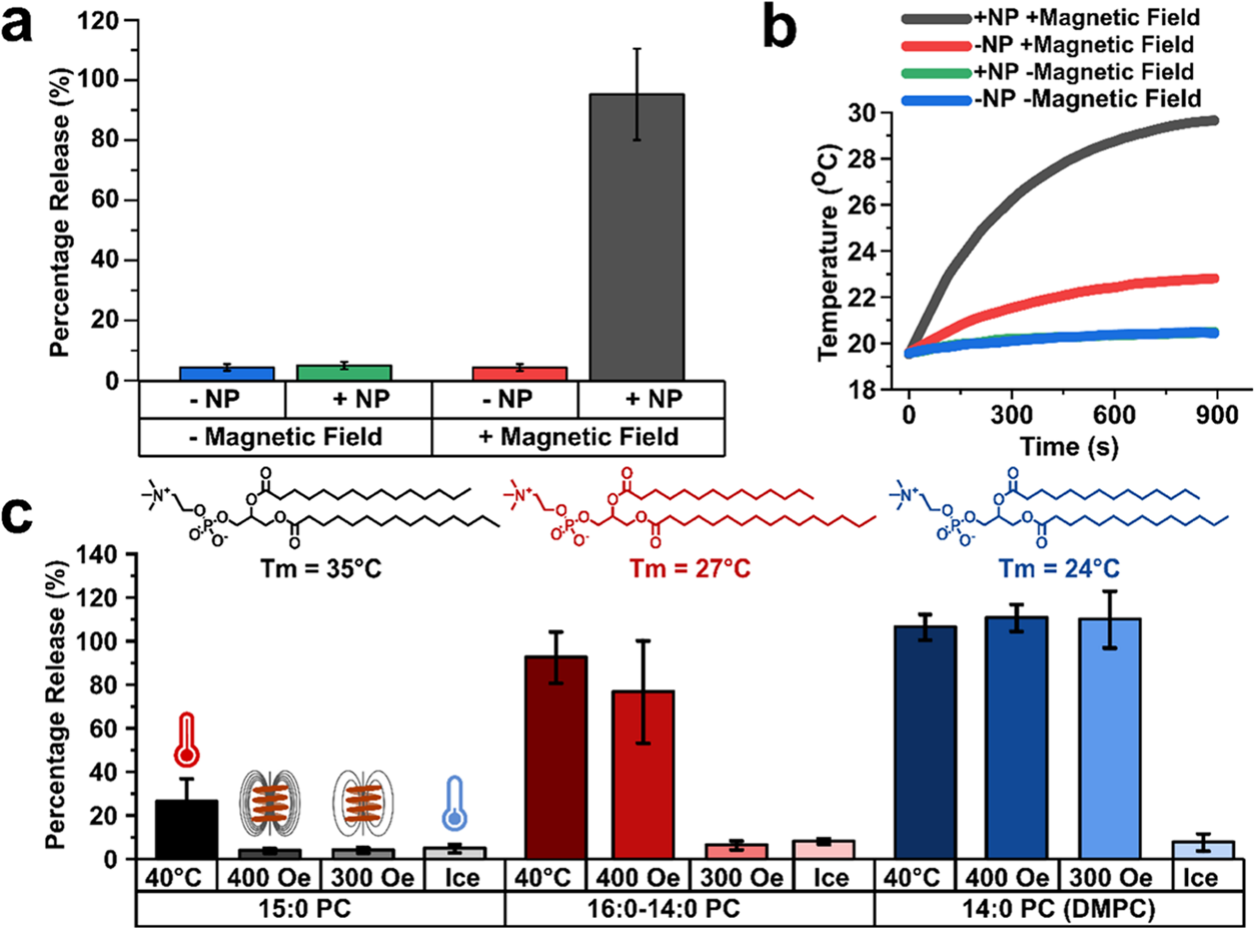
(a) Fluorescence intensity obtained from calcein release from 200 nm DMPC organelles with and without the addition of 2.5 mg/mL magnetic nanoparticles and a 400 Oe magnetic field. A large increase in fluorescence was only observed when both the magnetic nanoparticles and magnetic field were present. 100% is defined as the intensity achieved after lysis with the surfactant Triton X-100. (b) Graph showing the increase in temperature as the 200 nm synthetic organelles are exposed to the magnetic field with and without the magnetic nanoparticles (in bulk; not confined in GUVs). There is a small ∼2 °C increase within the non-nanoparticle sample exposed to the magnetic field (red, conduction heating), while there is a large increase observed in the sample with both magnetic nanoparticles and magnetic field (black, induction heating). (c) Graph showing how the modification of the lipid composition can affect the strength of the magnetic field needed to cause release of contents of the 200 nm organelles. Larger transition temperatures and longer chain lengths require larger magnetic field strengths to cause release.

Bulk experiments were performed to determine the degree of release obtained from the 200 nm synthetic organelles. The self-quenching dye calcein was used to perform these experiments to better understand the specific role of the release from the 200 nm synthetic organelles within the system. Nonstreptavidin-coated nanoparticles (2.5 mg/mL) were used in these experiments to allow for easy removal of the nanoparticles, which impact the fluorescence due to their dark hue (SI Figure 6). Four conditions were used for the 200 nm synthetic organelles to probe two defining variables: the impact of the magnetic field and the impact of the presence of the nanoparticles. As can be seen in Figure 3a, without the presence of the nanoparticles (red), there was little to no impact on the calcein release upon the application of the magnetic field; however, there was a considerable change in the samples containing nanoparticles (black). It can therefore be determined that the calcein release observed is dependent on both the presence of nanoparticles and exposure to the magnetic field; content release and biochemical activation was therefore not due to direct conductive heating from the coil itself.

To further understand the temperature characteristics of our system, we performed experiments with our organelles in bulk (i.e. not encapsulated in GUVs) and measured the temperature of the solution under various conditions (Figure 3b). In the presence of both NPs and a magnetic field, a ∼10°C temperature increase was recorded. In the absence of a magnetic field, only a minimal (∼0.5°C) temperature increase was observed, which was attributed to the continued equilibration of the samples to room temperature after being kept on ice. When the magnetic field was present in the absence of NPs, a ∼2°C increase in temperature was observed. This increase, attributed to conductive heating, was not sufficient to cause content release from our thermoresponsive vesicles (further confirmed by the results in Figure 3a). These results confirm that release from our organelles, and syntehtic cell activation, was due to magnetic induction heating.

Further modulation of this release could be controlled through engineering the lipid membrane and the field strength of the magnet. Three different lipid compositions (14:0 PC, 16:0–14:0 PC, and 15:0 PC) with phase transitions of 24, 27, and 35 °C, respectively,^55^ were used to form calcein-loaded synthetic organelles. These organelles were then mixed with nanoparticles before being exposed to a magnetic field for 20 min (Figure 3c). DMPC synthetic organelles were found to release at both 300 and 400 Oe, which agreed with the results observed in the synthetic cell experiments. At 300 Oe, a similar degree of release to 400 Oe was observed in these bulk experiments, which differed from the synthetic cell experiments and can be attributed to the higher concentration of NPs (2.5 mg/mL) present in the bulk experiments compared to those encapsulated within the synthetic cells (<2.5 mg/mL, due to low encapsulation efficiency). At 400 Oe, leakage was also observed in the 16:0–14:0 PC organelles that was not previously present at 300 Oe. Meanwhile, minimal leakage was observed at both these magnetic field strengths in the 15:0 PC sample, indicating that the heating induced by the nanoparticles was below the transition temperature of the 15:0 PC membranes. These experiments confirm that we can couple membrane composition with the applied magnetic field strength to control the initiation of nanoparticle-mediated content release from the synthetic organelles.

This system could be adapted for lipids with higher gel–fluid phase transition temperatures by further optimizing the heating capability of the nanoparticles. The degree of heating within Fe_3_O_4_ nanoparticles is affected by many factors including anisotropy, magnetic susceptibility, and saturation magnetization, which are all dependent on each batch of nanoparticles.^33,34,43^ The lipid membranes could also be tuned through the addition of lyso-PC and poly(ethylene glycol) lipids (PEG-lipids) to stabilize the grain boundary defects formed at the phase transition, allowing for more efficient release from the synthetic organelles.^41,56^ These optimizations would allow for direct applicability within an in vivo context and open the possibility for the usage of this technology in biotechnology applications including remote-controlled multistage drug release systems or microreactors to produce unstable or toxic drugs at the target site.

## Conclusions

We have laid the foundations for future work in the area of magnetically inducible synthetic cells by establishing a proof of principle with enzymatic systems. This can be further developed to apply to more complex biochemical responses such as protein synthesis. The development of this technology in conjunction with the continual advancement of other applications of magnetic nanoparticles, e.g., in the field of directed movement, also allows for the future possibility of integrating these modalities to operate under a dual system, producing a synthetic cell which could be magnetically directed before magnetically inducing activation and release from the synthetic cell system.

## Supporting Information Available

Materials and methods; nanoparticle heating kinetics in pure water; DLS data of nanoparticles; image of the induction magnet setup; temperature change of 0.125 mg/mL nanoparticles within the magnetic field; representative microscopy images of synthetic cells; microscopy images of selected synthetic cells including on ice and heated; and photo comparison of fluorescence observed with and without nanoparticles (PDF)
