# Supplementary material for: Magnetic Modulation of Biochemical Synthesis in Synthetic Cells
Source: J Am Chem Soc. 2024 May 1;146(19):13176–82. doi: 10.1021/jacs.4c00845 (PMC11099998; doi:10.1021/jacs.4c00845)
Supplement: Supplementary file 1 — ja4c00845_si_001.pdf [file ja4c00845_si_001.pdf]

Magnetic Modulation of Biochemical Synthesis within Synthetic Cells

Karen K. Zhu [1,2,3,4], Ignacio Gispert Contamina [2,3], Oscar Ces [1,3,4], Laura M. C. Barter [1,4], James W. Hindley [1,3,4]
Yuval Elani [2,3*]

[1] Department of Chemistry, Imperial College London, Molecular Sciences Research Hub, White City, London W12 0BZ, UK
[2] Department of Chemical Engineering, Imperial College London, South Kensington, London, SW7 2AZ, UK
[3] fabriCELL, Imperial College London, Molecular Sciences Research Hub, White City, London W12 0BZ, UK
[4] Institute of Chemical Biology, Imperial College London, Molecular Sciences Research Hub, White City, London W12 0BZ, UK

1 Materials & Methods

All lipids were obtained from Avanti Polar Lipids, all nanoparticles were obtained from Ocean Nanotech, while all other chemicals were obtained from Sigma Aldrich unless otherwise stated.

1.1 Magnetic Heating of Nanoparticles with Temperature Monitoring

An EasyHeat 3542 was used to achieve induction heating with a field strength of 400 Oe, 400 kHz. Samples were generally placed within a magnetic field of 400 Oe for 30 minutes. The temperature change was monitored using an optical fibre temperature probe.

1.2 200 nm Synthetic Organelle Production for the Nested System

200 nm synthetic organelles were prepared via the thin-film hydration method with lipid mixtures formed from 25 mg/mL $CHCl_3$ stocks. Films of DMPC with 1% Biotinyl Cap PE 18:1 were produced by vigorously mixing the stocks and evaporating the CH_3Cl under a stream of N_2 (g) before further drying under vacuum for 12-15 hrs. The films were then hydrated with a fluorescein di-β-D-galactopyranoside solution (1.5 mM fluorescein di-β-D-galactopyranoside, 500 mM sucrose, 20 mM HEPES pH 7.4, 100 mM KCl) to lipid concentrations of 20 mg/mL respectively.

The organelles were then freeze-thawed 10 times; freezing with N_2 (l) before heating to 50 °C, before extrusion through a 200 nm polycarbonate filter, also performed at 50 °C. The unencapsulated contents were then removed via size-exclusion chromatography through a Sephadex G-50 column. The elution with sucrose buffer (500mM sucrose, 20 mM HEPES, 100 mM KCl) was performed in a cold room at 4 °C and the purified organelles kept on ice.

The samples without nanoparticles were then diluted in a 1:1 solution with more sucrose buffer (500mM sucrose, 20 mM HEPES, 100 mM KCl) while the samples that were intended to contain nanoparticles were diluted in a 1:1 solution with 5 mg/mL Fe_3O_4 30nm streptavidin-coated nanoparticles in a 500 mM of sucrose solution.

1.3 Nested Vesicle Synthetic Cell Production

The nested vesicles were prepared using the emulsion phase transfer method. A film of POPC was dissolved in mineral oil by sonication for 45 minutes to produce a 6 mg/mL solution of POPC. An inner aqueous solution of the FDG 200 nm synthetic organelles (with or without NP) in sucrose buffer and β-Gal 5 U/mL was prepared. An emulsion of the inner solution (20 µL) and the POPC-in-oil solution (200 µL) was produced through pipetting up and down repeatedly. This emulsion was then carefully layered onto 300 µL of glucose buffer (500 mM glucose, 20 mM HEPES, 100 mM KCl) and centrifuged at 4 °C at either 4000 g for 6 minutes for the samples without nanoparticles, or 1000 g for 30 s for the samples with nanoparticles. The oil layer above the aqueous layer was then carefully

removed via pipetting and 2 identical samples were combined and diluted to 800 µL for the testing of 4 conditions, and 4 were combined and diluted to 1600 µL for the testing of 7 or more conditions.

1.4 Induction-Heating Nested Synthetic Cells with the Magnet

The samples were then divided into separate conditions of 200 µL. With one sample kept on ice through all the proceedings, one placed within the magnetic coil to experience the temperature of the room and cooling system without the activation of the magnetic field (which was set to 18 °C), and some placed one at a time within the magnetic coil with the field activated at 100 Oe, 200 Oe, 300 Oe or 400 Oe (induction heating) and the last sample was heated in an Eppendorf thermocycler set to 40 °C. All samples (apart from the samples kept on ice) were allowed to reach the temperature of the coil (15 minutes at ambient temperature) before being exposed to their allocated environmental conditions for a further 15 minutes. The synthetic cells were then left to stand for 30 minutes on ice before analysis.

1.5 Image Analysis

5 fluorescent images were obtained for each of 3 repeats to obtain 15 images in total for each condition. These were obtained on a Nikon Ti2 microscope using a FITC cube, λ_{ex} = 490 nm. All the synthetic cells in each photo were located as regions of interest (ROIs) and the mean intensity of each region measured. The means were then normalised after each repeat of the experiment according to the following formula: $\frac{I-I_I}{I_H-I_I} \times 100\%$, with I being the each intensity, I_I being the average intensity achieved from the samples kept on ice, and I_H being the average intensity of the samples that were heated to 40 °C.

1.6 200 nm Synthetic Organelle Production and Usage for Bulk Experiments

The preparation of these 200 nm synthetics organelle was identical to that of the 200 nm synthetic organelles for the nested system however the films were made with DMPC, 16:0-14:0 PC or 15:0 PC respectively with Biotinyl Cap PE 18:1 and were hydrated with a calcein solution (50 mM calcein, 20 mM HEPES pH 7.4, 100 mM KCl) to lipids concentrations of 10 mg/mL. Once the organelles had been extruded 21 times, freeze-thawed 10 times and purified via size-exclusion chromatography the samples were similarly diluted 1:1 with sucrose buffer (500mM sucrose, 20 mM HEPES, 100 mM KCl) or nanoparticles (5 mg/mL), however these nanoparticles did not possess a streptavidin coating.

1.7 200 nm Synthetic Organelles in Bulk Calcein Release Assay

Two 200 µL fractions of purified organelles were left without nanoparticles, while two had nanoparticles. 1 of each of these conditions was then left at ~18 °C for 30 min, while the other set was left at ~18 °C for 15 min before being exposed to the magnetic field of strength 400 Oe for 15 min. The samples were then left to sit on ice for 30 min before being centrifuged at 9000 g for 10 min to pellet the nanoparticles and allow for removal of the supernatant.

For the modulation experiments all samples had nanoparticles added and were left at ambient temperature ~18 °C for 15 minutes before being kept at 300 Oe, 400 Oe or 40 °C for 15 minutes. The negative controls were kept on ice for the whole duration. The samples were then centrifuged at 9000 g for 10 min to pellet the nanoparticles and allow for removal of the supernatant.

The fluorescence of the supernatants was then obtained from a CARY Eclipse Fluorescence Spectrometer (Agilent) at $\lambda_{ex/em}$= 495/515 nm before and after the addition of 1 µL of 5% Triton X-100.

A normalisation was then performed using the equation: $\frac{I}{I_T} \times 100$. Where I is the intensity of the sample, and I_T is the intensity achieved after the addition of Triton X-100.

1.8 Nanoparticle Dilutions

5 mg/mL 30 nm Fe_3O_4 nanoparticles were diluted to concentrations of 2.5, 1.25 and 0 mg/mL and exposed to a 400 Oe, 400 kHz magnetic field for 30 minutes and the change in temperature was recorded using and optical fibre temperature probe.

1.9 Size determination of Organelles

A Zetasizer (Malvern) was used to determine the size distribution of the organelles using dynamic light scattering (DLS).

1.10 Determining the Bulk Change in Temperature for 0.125 mg/mL Fe_3O_4 Nanoparticles

The temperature of a sample of 0.125 mg/mL Fe_3O_4 nanoparticles in water was measured using a thermocouple before and immediately after 15-minute application of a 400 Oe, 400 kHz magnetic field.

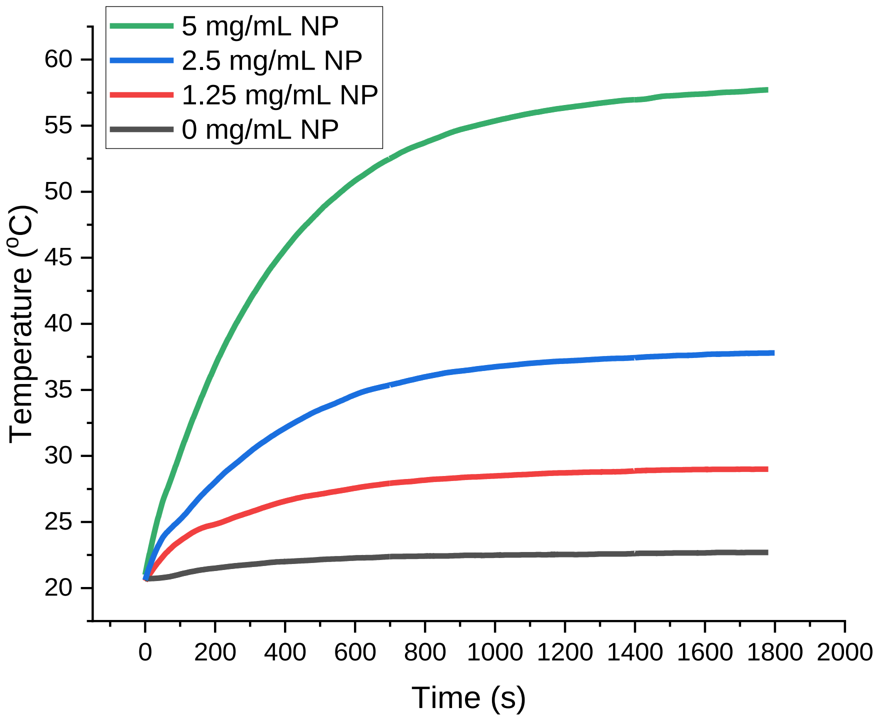

SI Figure 1: Heating kinetics of Fe_3O_4 nanoparticles in an alternating magnetic field (400 Oe, 400 kHz) for 30 minutes in bulk.

Conditions include: 5 mg/mL undiluted nanoparticles; 2.5 mg/mL 1:1 dilution of nanoparticles in H_2O; 1.25 mg/mL 1:3 dilution of nanoparticles in H_2O; 0 mg/mL pure water. Maximum temperature recorded was 58 °C , 37 °C, 28 °C and 22 °C respectively. Slight increase in temperature of 0 mg/mL due to conduction heating from the coil.

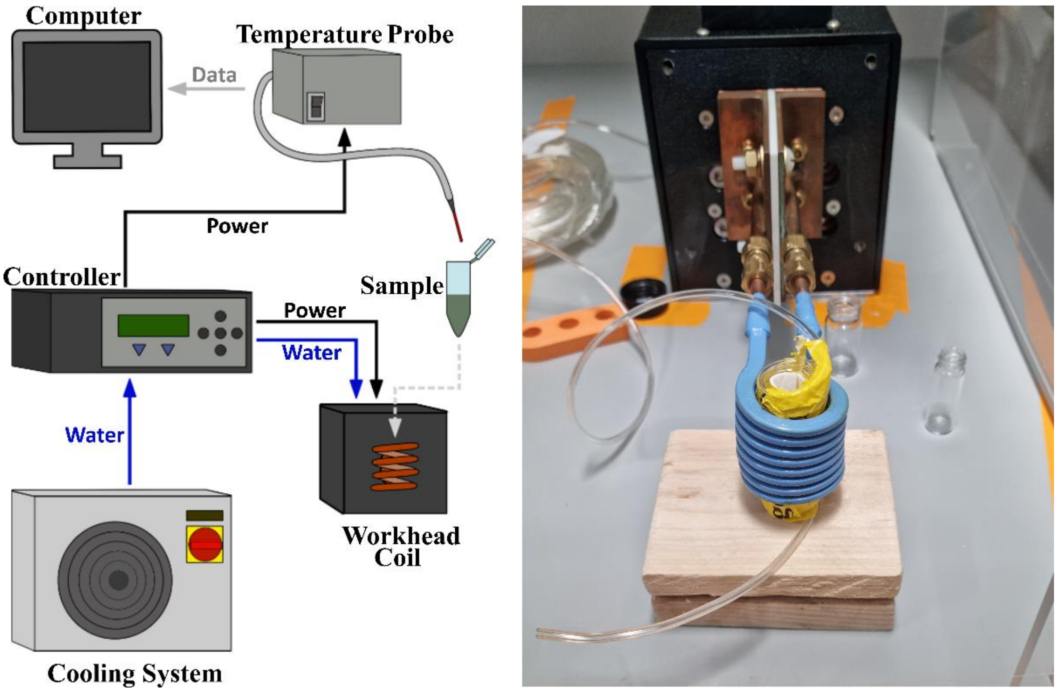

SI Figure 2: Schematic of Equipment (Left), Photo of Workhead coil (Right).

Insulation added to workhead coil: 1 layer of fibreglass insulation, and 1 coil of pipe through which compressed air is flowed.

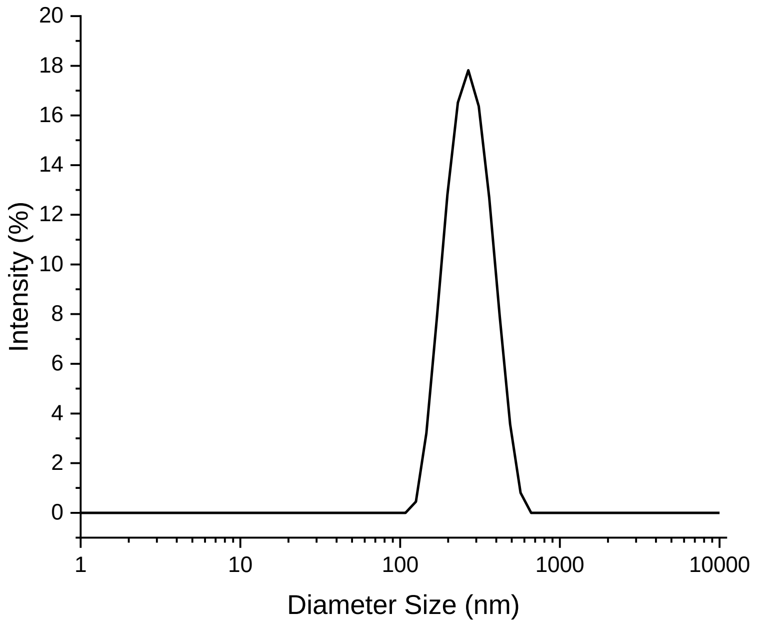

SI Figure 3: Dynamic Light Scattering (DLS) of 200 nm DMPC vesicles containing FDG

Temperature Change with 0.125 mg/mL nanoparticles	
Starting Temperature (°C)	19.7
Temperature after 15 mins at 400 Oe (°C)	22.3
Temperature after 15 mins 0 mg/mL NP	22.8

SI Table 1: Temperature of 200 µL, 0.125 mg/mL NP after exposure to 400 Oe for 15 mins.

To further demonstrate that our system activation was due to localized heating within the GUVs, rather than bulk heating, we performed the following experiment. First we assumed an extreme scenario where all GUVs ruptured, releasing all contained NPs into the bulk, resulting in a dilution to 0.125 mg/mL. We then measured the temperature increase of the solution at this concentration, as shown in the table above. After 15 minutes at 400 Oe, the temperature in a 0.125 mg/mL nanoparticle concentration in 500 mM sucrose buffer did not significantly differ from the maximum temperature achieved by a 0 mg/mL NP concentration under the same magnetic field conditions. This indicates that induction heating was minimal, and confirms that nested GUV activation was due to localized heating within the GUVs rather than bulk heating of the sample.

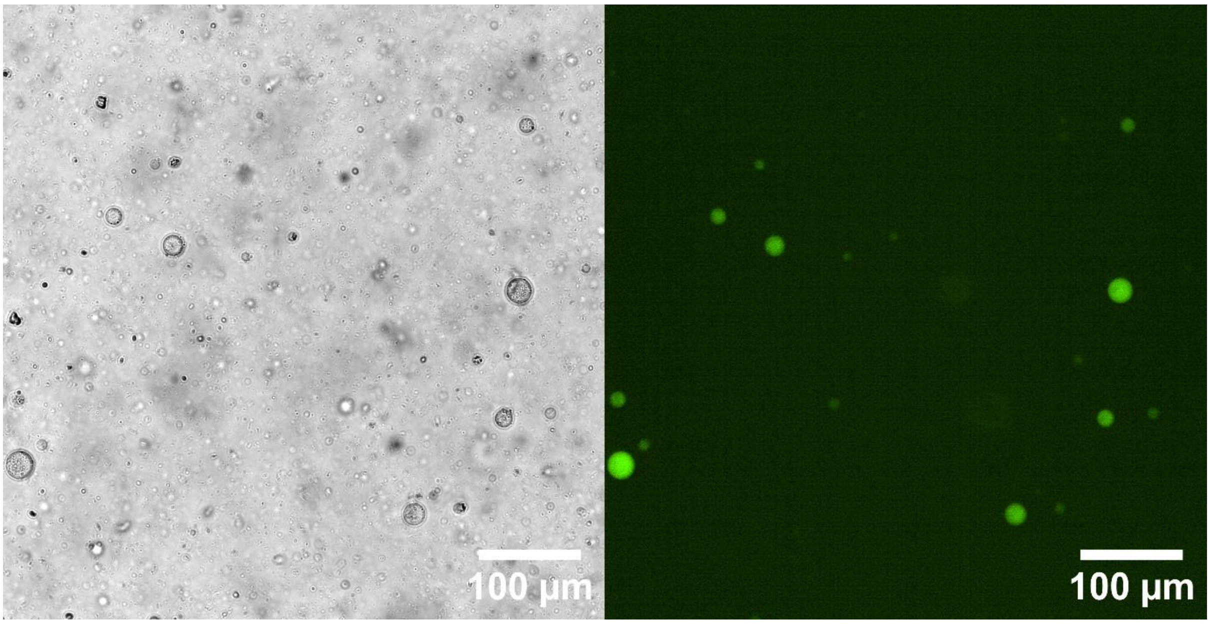

SI Figure 4: Complete field view of the nested system after exposure to a 400 Oe magnetic field. Bright Field (left) and FITC filter fluorescence (right).
Most GUVs that formed contained sufficient 200 nm vesicles with FDG, nanoparticles (NP), and β-Galactosidase (β-Gal) to produce a visible fluorescein signal.

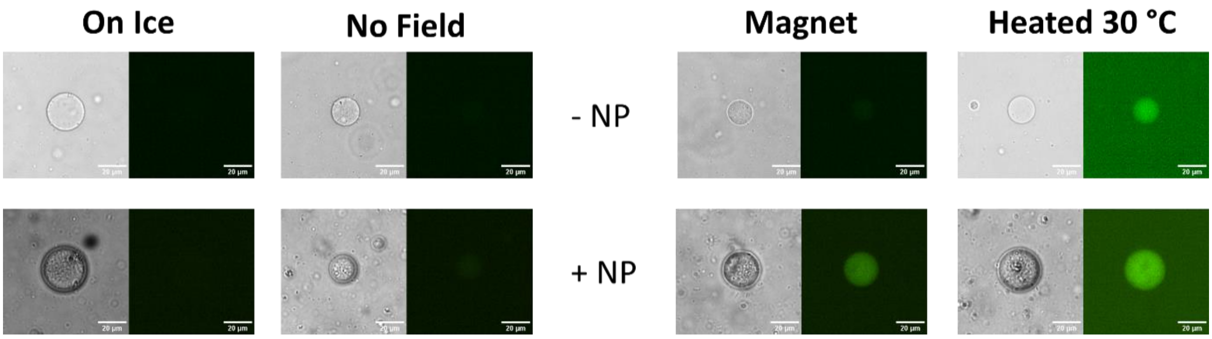

SI Figure 5: Representative microscopy images of vesicles under various conditions*.*
A clear increase in fluorescence was observed in the presence of both the magnetic field and nanoparticles. Samples, both with and without nanoparticles, showed no fluorescence when kept on ice. However, they exhibited the highest fluorescence when heated to 30°C. This heating forced the 200 nm DMPC vesicles to undergo a phase transition, releasing FDG to produce fluorescein, which in turn caused the observed fluorescence.

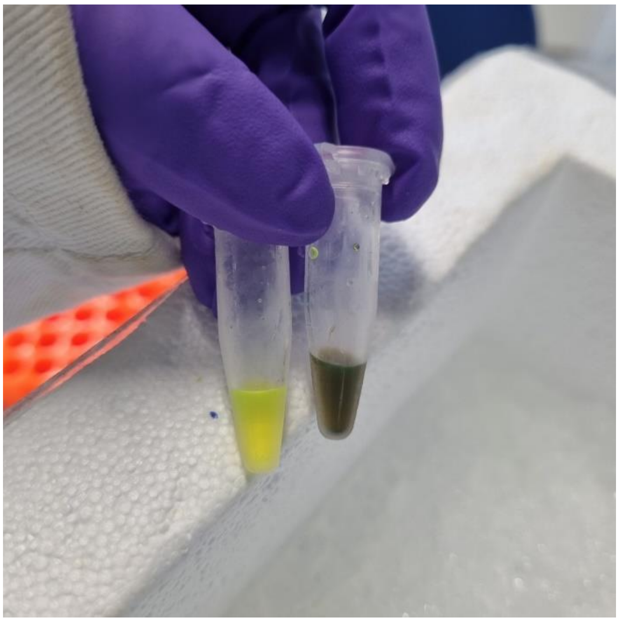

SI Figure 6: 200 nm vesicles after calcein release with and without nanoparticles

The dark hue and spectral adsorption of the nanoparticles causes interference of the fluorescent signal. Hence, nanoparticles were removed prior to fluorimetry recordings in the bulk release experiments.
